# Circadian rhythm and sleep alterations in older people with lifetime depression: a case-control study

**DOI:** 10.1186/s12888-020-02606-z

**Published:** 2020-04-29

**Authors:** Camilla M. Hoyos, Christopher Gordon, Zoe Terpening, Louisa Norrie, Simon J. G. Lewis, Ian B. Hickie, Sharon L. Naismith

**Affiliations:** 1 The University of Sydney, Faculty of Science, School of Psychology, Sydney, NSW Australia; 2 The University of Sydney, Brain and Mind Centre, Healthy Brain Ageing Program, Sydney, NSW Australia; 3 CIRUS, Centre for Sleep and Chronobiology, Woolcock Institute of Medical Research, The University of Sydney, Sydney, Australia; 4 The University of Sydney, Faculty of Medicine and Health, Susan Wakil School of Nursing and Midwifery, Sydney, NSW Australia; 5 Faculty of Medicine, University of New South Wales, Sydney, Australia; 6 St Vincent’s Hospital Older People’s Mental Health Service, Darlinghurst, NSW Australia

**Keywords:** Depression, Older adults, Sleep-wake disturbance, Circadian phase, Circadian misalignment

## Abstract

**Background:**

Depression is common in older people and is associated with underlying brain change increasing the risk of dementia. Sleep disturbance is frequently reported by those with lifetime depression, however whether circadian misalignment also exists is unclear. We aimed to examine circadian rhythms and sleep associations in older patients with and without lifetime depression.

**Methods:**

Thirty-four older people meeting DSM-IV criteria for lifetime major depression (mean age = 63.9 years), and 30 healthy controls (mean age = 65.7 years) were recruited. Participants underwent 2-weeks of actigraphy followed by a 3-night protocol including dim light melatonin onset (DLMO) assessment and overnight polysomnography (PSG) for sleep architecture. DLMO and phase angle of entrainment were computed.

**Results:**

Compared to controls, participants with depression had a significantly longer phase angle of entrainment (6.82 h ± 1.45 vs. 5.87 h ± 1.60, *p* = 0.02, Cohens-d = 0.62). A small to moderate yet non-significant difference in DLMO times, with earlier DLMO (34 ± 27 min) observed in depression (20:36 ± 1:48 vs. 21:10 ± 1:48, *p* = 0.22, Cohens-d = 0.32). Individuals with depression had longer sleep latency and latency to rapid eye movement sleep than controls (all *p* < 0.05).

**Conclusion:**

Circadian advancement and alterations to the timing of sleep and REM onset are evident in older people with lifetime major depression, despite having only mild residual symptoms. Further research examining the prognostic significance of these changes is warranted as well as chronotherapeutic treatment studies.

## Background

Within the increasing ageing population, depression in older people will continue to be a predominant health care problem. This syndrome is associated with significant disease burden, disability, functional decline and premature death [1]. Depression increases burden on caregivers, health services and is associated with increased health care expenditure [2]. Of significance, depression in older people is associated with cognitive impairment [3], disability [4] and progression to dementia longitudinally [5, 6]. Even sub-threshold depressive symptoms carry a significant risk for subsequent onset of major depression [7], ongoing cognitive decline [8] and dementia [9], warranting efforts focused on delineating modifiable risk factors. One potential candidate in this regard, is sleep and circadian (sleep-wake) disturbance.

The significance of sleep-wake functions for mood and cognition is underscored by a number of clinical, epidemiological and longitudinal studies showing that sleep-wake disturbance is a prodromal feature of depressive symptom onset, that it may persist in the remitted state and that it may perpetuate the illness [10–13]. Insomnia and depression are highly co-prevalent and likely to be mutually precipitating [14]. In older people with depression, insomnia is the most commonly reported sleep disturbance; with complaints of early morning wakefulness being common [15, 16]. Polysomnography (PSG) studies show alterations in slow wave sleep (SWS) and rapid eye movement (REM) sleep as well as increased latency to REM sleep, increased wake after sleep onset and reduced sleep efficiency [17], see review by [18]. Sleep disturbances in patients with depression are indicative of more severe illness and tend to be under-treated [19]. In older people with depression, sleep disturbance may be most prominent at the onset of a depressive episode [20–22] and is predictive of treatment responsiveness [23]. Thus, the presence of this feature has considerable clinical and prognostic significance.

To date, examination of sleep-wake patterns in older people with depression has focused largely on sleep, without concurrent examination of the circadian system. Given the increasing recognition of the integral role of circadian change in depressive disorders [24], studies examining these two systems concurrently are warranted. Whilst it is clear that circadian phase advancement occurs during healthy ageing with a concomitant reduction in circadian amplitude [14], it is not clear whether patients with lifetime major depression exhibit similar circadian patterns and/or whether the sleep and circadian changes are more pronounced. Certainly in middle-aged samples and in those with seasonal affective disorder, some [25–29] but not all [30] evidence suggests alterations in melatonin amplitude and rhythm and circadian changes may occur at the onset and maintenance of affective disorders [25]. In younger samples, there also appears to be a phase delay in circadian timing, and melatonin secretion may be altered even in very early stages of a depressive illness [24, 31]. However, there is some evidence to suggest that circadian alterations in affective disorders may vary according to the ageing process, with younger patients showing the greatest phase delay, and older patients showing lower circadian amplitude [32].

A greater understanding of the changes to the circadian system in older people with depression is not only of immense scientific interest, but importantly this information could inform personalised treatment approaches for both depressive symptomatology and cognitive decline [24, 33]. In this study, we aimed to examine circadian rhythms in older patients with depression, with the primary outcomes of interest being dim light melatonin onset and phase angle of entrainment. Secondary outcomes were measurements of sleep architecture and quality assessed both objectively and subjectively. We also sought to examine if there were associations between clinical correlates and circadian rhythmicity in an exploratory analysis to understand possible future clinical and therapeutic targets. We hypothesized that relative to controls, older patients with depression would show phase advancement and greater sleep disturbance.

## Methods

### Participants

Health-seeking older adults were recruited from specialist psychiatry clinics at the Brain & Mind Centre at the University of Sydney, Sydney, Australia. To be eligible participants had to: be aged 50 years or older; meet DSM-IV criteria for lifetime major depressive disorder; have had a depressive episode within the last 5 years; and be clinically stable on medication. Exclusion criteria were: other psychiatric illness including bipolar disorder; history of stroke; neurological disorder; head injury with loss of consciousness > 30-min; medical conditions known to affect cognition (e.g. cancer); diagnosis of dementia and /or Mini Mental State Examination Score < 24 [34]; current shift-workers; transmeridian travel within the prior 60-days; use of medication that may affect sleep and/or melatonin secretion such as beta-blockers or lithium. Individuals taking sedative hypnotics including benzodiazepines were asked to stop the medications for 2 weeks prior to the study visit. Sleep apnoea was excluded based on clinician interview and Apnoea Hypopnoea Index score when available. Age-matched control participants were from the community and had to not have any history of lifetime psychiatric disorders or any of the above mentioned exclusion criteria. Participants needed to agree to wear an actigraphy watch and complete sleep diaries for 2 weeks as well undertake overnight PSG and circadian assessments on three consecutive nights within the research facility. This research was approved by the Human Research Ethics Committee of The University of Sydney and was conducted in accordance with the latest version of the Declaration of Helsinki. Written informed consent was obtained from all participants.

### Procedure

Participants were first screened over the telephone and then attended a screening visit to confirm study eligibility. Prior to attending the three-night protocol, participants were asked to wear an actigraph (Actiwatch Spectrum, Philips Respironics, OR) and complete a sleep diary for 2 weeks.

### Measures

#### Psychiatric

As detailed elsewhere, a structured clinical assessment was conducted by an Old Age Psychiatrist, which included psychiatric history, depression onset age, risk of sleep disorders, body mass index and medication use. Medical burden was quantified using the Cumulative Illness Rating Scale, Geriatric Version [35]. Lifetime and current depression were confirmed using the affective component of the Structured Clinical Interview for DSM-IV-R [36]. The Hamilton Depression Rating Scale was used to determine depression severity [37].

#### Sleep and circadian measures

i) *Melatonin sampling:* Salivary melatonin was measured to determine DLMO on the third night of the protocol, as detailed previously [31, 38]. Participants arrived 7 h prior to their Habitual Sleep Onset (HSO: determined from 14-day actigraphy and/or sleep diary) and were kept awake until 2 h after HSO time. Saliva samples (1.5 ml, using Salivette, Sarstedt, Germany) were collected 30-minutely (6-h prior to until 2-h after HSO). Participants maintained an upright seated posture and refrained from food or drink for at least 20-min prior to each saliva sample collection. Samples were immediately frozen at -20 °C. Melatonin was assayed using 200ul of saliva by double antibody radioimmunoassay (Cat# RK-DSM2; Buhlmann Laboratories AG, Schönenbuch, Switzerland). The lowest detectable level of melatonin was 1 pg/mL (4.3pM). The DLMO timing was calculated when melatonin levels passed and remained above the absolute threshold of 3 pg/mL (12.9 pM) and calculated using linear interpolation between successive 30 min saliva samples below and above the threshold for each subject [39]. The phase angle of entrainment, a measure of the relationship between the biological clock and a recurring external cue, was calculated by subtracting DLMO time from the midpoint of sleep determined from actigraphy (hh:mm) [40].
ii) Self-report questionnaires: This included the Pittsburgh Sleep Quality Index to assess sleep quality over the previous month. The scale ranges from 0 to 21 with a higher value indicating greater sleep disturbance. A score > 5 is defines as impaired sleep quality [41].
iii) Actigraphy monitoring: Participants were asked to wear an actigraphy watch and keep a sleep diary, under usual light and behavioural conditions, for 14 days prior to commencing the in-laboratory portion of the experimental protocol. All procedures have been previously described [42]. Habitual sleep onset, total sleep time and sleep midpoint were used to assess the participants day-to-day sleep patterns.
iv) Polysomnography: Participants attended the Chronobiology and Sleep Laboratory at The Brain & Mind Centre for three consecutive nights. On the first two nights, PSG recordings were collected using an ambulatory recording system (Compumedics Siesta, Australia). Night one was considered an acclimatization night and included a clinical assessment to detect the presence of occult sleep disorders, such as obstructive sleep apnea syndrome, periodic limb movements, or restless legs syndrome. Sleep variables collected on night two, using a standardised research PSG montage (electroencephalogram, electrooculogram, electromyogram) were used for all analyses. Sleep architecture stages were visually scored in a computer using standardised criteria by an experienced sleep technician [43], with modifications for older participants [44]. Laboratory conditions were controlled with fixed lighting levels (< 30 lx during waking; < 10 lx during DLMO testing; < 1 lx during scheduled sleep periods) and ambient temperature (24 ± 1 °C). Additionally participants were physiologically and behaviourally monitored at all times. Latency to REM sleep (time from sleep onset, as defined by three contiguous epochs of stage 1 sleep, or any other stage of sleep to the first epoch of REM sleep [mins]), wake after sleep onset (mins) and the number of arousals (defined as an abrupt shift in electroencephalogram frequency of 3 seconds or longer; arousals during rapid eye movement sleep required an increase in chin electromyogram activity) were derived from the PSG. For descriptive purposes we also report the time of sleep onset (24-h clock time); latency to sleep (time from ‘lights out’ to sleep onset, as defined by three contiguous epochs of stage 1 sleep, or any other stage of sleep [mins]); the time spent in slow wave sleep (sum of stage 3 and stage 4 sleep) (mins), non-REM sleep and REM sleep (mins); sleep efficiency (total sleep time-latency to sleep onset/time in bed*100, %); and, the apnoea-hypopnoea index (number of apnoea plus hypopnoea events per hour of sleep).

### Statistical analysis

We undertook purposive sampling for cases and controls from the Healthy Brain Ageing clinic database. All data are reported as means and standard deviations unless otherwise stated. All analyses were conducted using IBM® SPSS® Statistics Version 25 Chicago: IBM Inc.. Between group comparisons were performed using independent samples t-tests, Mann-Whitney U tests or Chi-squared tests where warranted. Normality of outcome variables was determined by visual inspection of histograms and then Pearson and Spearman correlation coefficients were used as appropriate. Due to the small amount of missing data, data imputation methods were not utilised. All analyses were two-tailed and employed an alpha level of 0.05.

## Results

**Fig. 1:**
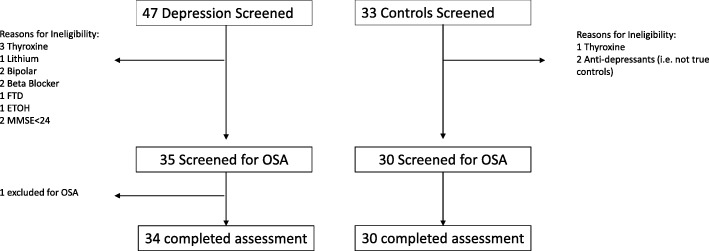
Study flow

Forty-seven cases and 33 controls were screened for the study. Thirty-four participants with lifetime depression and 30 controls met eligibility criteria. Reasons for ineligibility are shown in the study flow (Fig. 1). In the cases, the mean age of depression onset was 42.2 ± 19.1 years (range 10 to 82 years), with the median number of depressive episodes being 2.0 (inter-quartile range 1.0–3.0). Nine participants met DSM-IV criteria for current major depression at the time of assessment. Twenty-three participants were taking antidepressant medications (tricyclics *n* = 6; Selective Serotonin Reuptake Inhibitors [SSRIs] n = 6; Serotonin Norepinephrine Reuptake Inhibitors [SNRIs] *n* = 8; mirtazapine *n* = 2; Noradrenaline Reuptake Inhibitors [NRI] *n* = 1).

**Fig. 2:**
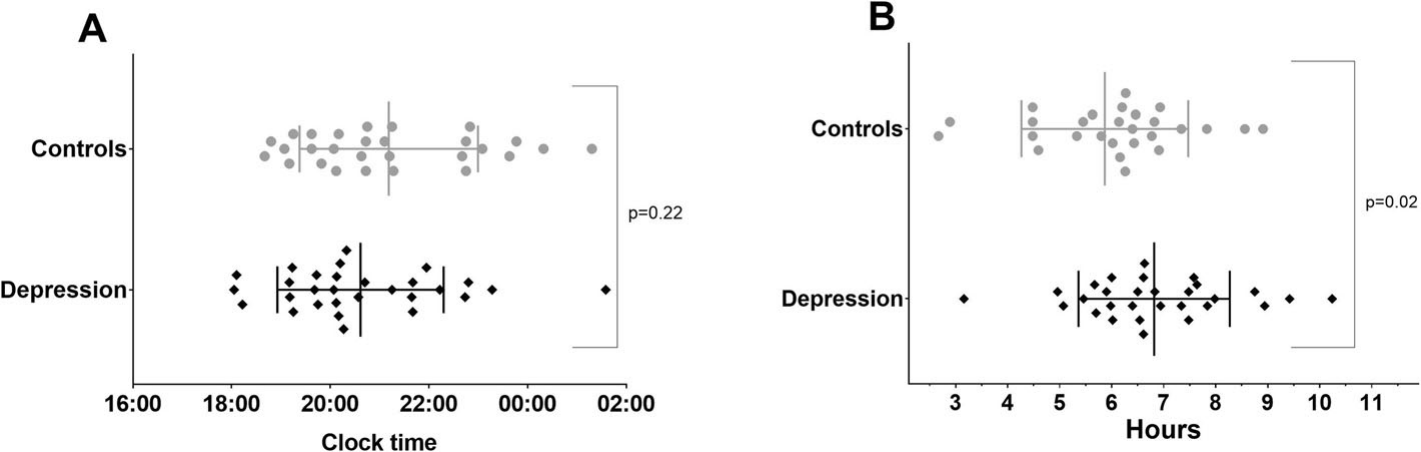
Differences in dim light melatonin onset times (A) and phase angle between dim light melatonin onset time and midpoint of sleep (B) between depression cases and controls. Grey circles are controls and black diamonds are depression patients. Points are the individual values, the middle line is the mean and the error bars are the standard deviation

As shown in Table 1, there were no significant differences between cases and controls in terms of age, gender, years of education, global cognition (Mini Mental State Examination Score scores), body mass index or level of medical illness burden (Cumulative Illness Rating Scale). As expected, cases had significantly higher levels of depressive symptoms than control participants as evidenced by scores on the Hamilton depression rating scale and geriatric depression scale. Those with depression had poorer self-reported sleep quality as assessed by the Pittsburgh sleep quality index (Fig. 2).

**Table 1:** Demographic, psychiatric and sleep laboratory-based memory data for patients with depression (*n* = 34) and control participants (*n* = 30)

	Cases *n* = 34	Controls *n* = 30	Test statistic	Effect Size	*p*-value
Age, years	63.85 (10.76)	65.70 (9.11)	−0.74	0.18	0.46
Gender, male:female ^#^	17:17	17:13	−0.28	0.035	0.59
CIRS, total score ^‡^	4.00 (1.00, 6.00)	2.00 (1.00, 4.00)	1.81	0.24	0.07
HDRS, total score ^‡^	7.00 (4.00, 11.75)	1.50 (0, 4.00)	4.32	0.58	0.000
Geriatric Depression Scale, /30 ^‡^	17.00 (9.00, 22.50)	2.00 (0, 5.00)	5.51	0.71	0.000
PSQI, total score	8.30 (4.12)	5.07 (3.57)	3.3	0.84	0.002
Body Mass Index	25.5 (3.7)	27.1 (3.9)	−1.6	0.42	0.11
Education, years	13.5 (3.2)	13.7 (2.9)	−0.23	0.10	0.82
MMSE, /30^‡^	29.00 (27.00, 30.00)	30.00 (28.50, 30.00)	−1.58	0.21	0.11
WTAR, predicted IQ	106.3 (10.6)	107.2 (8.5)	−0.36	0.09	0.72
Depression Age of Onset (years)	42.2 ± 19.1 years	–	–	–	–
Median no. depressive episode	2.0 (1.0–3.0)	–	–	–	–

All test statistics are students t-test unless otherwise specified; ^‡^ Mann-Whitney U test *Z*-statistic #Pearson chi-squared statistic. Effect size is a calculated Cohen’s D and or using the formula Z-statistic/√N where appropriate. Data are mean (standard deviation), median (interquartile range) or n (%) as specified. *CIRS* Cumulative Illness Rating Scale; *HDRS* Hamilton Depression Rating Scale; *PSQI* Pittsburgh Sleep Quality Index; *MMSE* Mini Mental State Examination; *WTAR* Wechsler Test of Adult Reading; *ASS* age-scaled score

### Differences in circadian measures between groups

DLMO could not be computed for five cases and two controls due to inability to detect melatonin within the sensitivity of the assay or not reaching the 3 pg/mL threshold. There was no statistical difference in DLMO times between groups however, cases were found to be advanced, on average, by 36 min (± 25) compared to the control groups (Table 2 and Fig. 2). The phase angle of entrainment was significantly longer in cases compared to controls (Fig. 2 and Table 2).

**Table 2:** Circadian, actigraphy and sleep polysomnography data for patients with depression and control participants

	Cases	Controls	Test statistic	Effect Size	*p*-value
*Circadian*	*n* = 29	*n* = 28			
Dim Light Melatonin Onset, time	20:36 (1:48)	21:10 (1:48)	−1.23	0.32	0.22
Phase angle sleep midpoint, hh:mm	6:46 (1:27)	5:52 (1:36)	2.34	0.62	0.02
*Actigraphy*	*n* = 34	*n* = 22			
Habitual sleep onset (hh:mm)	23:13 (1:24)	23:08 (1:05)	0.23	0.07	0.82
Habitual sleep offset (hh:mm)	7:45 (1:15)	7:06 (0:49)	0.42	0.61	0.02
Total sleep time (mins)	452.9 (58.2)	428.2 (58.1)	1.32	0.42	0.19
Sleep Midpoint (hh:mm)^‡^	3:01 (2:42, 3:49)	2:59 (2:31, (3:34)	1.18	0.17	0.35
*Polysomnography*	*n* = 26	*n* = 29			
Sleep onset, time (hh:mm)	23:14 (1:14)	23:01 (0:49)	0.76	0.20	0.46
Sleep offset, time (hh:mm)	7:13 (1:15)	6:47 (1:01)	1.42	0.39	0.16
Total sleep time, mins	375.5 (79.9)	388.9 (54. 1)	−0.72	0.20	0.48
NREM sleep, mins	297.2 (55.4)	304.2 (47.5)	−0.50	0.14	0.62
REM sleep, mins	78.3 (38.0)	84.7 (25.5)	−0.73	0.20	0.47
Slow Wave Sleep, mins	54.9 (46.5)	59.0 (35.4)	−0.37	0.10	0.71
Sleep Efficiency, %	75.4 (15.5)	80.7 (10.4)	−1.48	0.20	0.15
Latency to Sleep Onset, mins ^‡^	17.5 (8.4, 28.3)	8.5 (3.8, 21.8)	2.00	0.26	0.046
Latency to REM sleep, mins ^‡^	126.0 (89.5, 183.8)	65.5 (44.8, 77.3)	3.93	0.53	0.000
WASO, mins ^‡^	79.8 (38.6, 150.8)	70.0 (42.8, 109.5)	0.91	0.12	0.36

All test statistics are students t-test unless otherwise specified; ^‡^ Mann-Whitney U test *Z*-statistic. Effect size is a calculated Cohen’s D and or using the formula Z-statistic/√N where appropriate. *WASO* wake after sleep onset; *REM* rapid eye movement

### Correlates of phase angle in people with depression

In cases, phase angle was not significantly correlated with clinical measures including age (*r* = − 0.06, *p* = 0.75), age of onset of depression (*r* = 0.14, *p* = 0.41), depression severity as measured by the Hamilton depression rating scale (rho = − 0.07, *p* = 0.72), subjective sleepiness (Pittsburgh sleep quality index, *r* = 0.29 *p* = 0.13), body mass index (*r* = − 0.29, *p* = 0.14) or global cognition (rho = 0.26, *p* = 0.21). There was no difference in phase angle (mean difference 0.14 h; 95%CI − 0.86 to 1.13), DLMO (0:59 h; − 1:33 to 0:54) between those with depression who did and did not take antidepressant medication nor were there any differences between those who had current major depression (*n* = 9) and those with a history of depression (Phase angle: mean difference − 0.08; 95%CI − 1.59 to 1.42; DLMO 0:14 h, 95%CI − 1:05 to 1:34).

Table 2 shows that there were no differences in actigraphic measured pre-study sleep durations or sleep onset times between the depression and control groups. Furthermore, there were no significant differences between patients and controls in sleep onset time, total sleep time and wake after sleep onset using PSG. Habitual sleep offset was later in individuals with depression compared to controls which was driven by three people who were late sleepers. After removal of these three late sleepers the difference in the offset had gone but the effects observed in the circadian measurements remained.

**Fig. 3:**
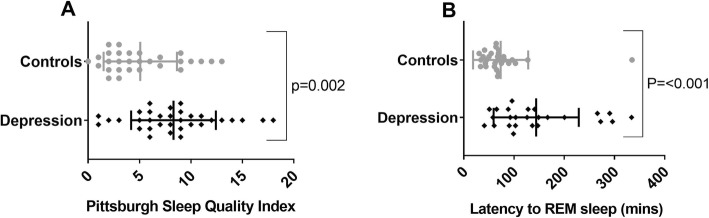
Differences in subjective sleep quality assessed using Pittsburgh Sleep Quality Index (**a**), and latency to REM sleep (**b**) measured using polysomnography between depression cases and controls. Grey circles are depression patients and black diamonds are controls. Points show individual values, middle line is the mean and error bars are the standard deviation

In comparison to control subjects, those with depression demonstrated longer latency to sleep onset and latency to REM sleep times (Table 2 and Fig. 3). There was no difference in the apnea hypopnea index, arousal index or number of arousals between the two groups. There was no difference between REM latency (z statistic, − 1.40, *p* = 0.18) between those with depression who did (median [IQR] = 136 min [91.5, 269.25]) and did not (99.5 min [65.625, 137.625]) take antidepressant medication.

## Discussion

This study shows for the first time that older people with largely remitted lifetime depression have a longer phase angle of entrainment relative to healthy controls, suggesting that the relationship between melatonin onset and sleep is misaligned. Indeed, DLMO was 34 min earlier on average in the depression cases, which corresponds to a small to medium effect size difference. This may be clinically relevant in this population; however, future studies are required to confirm. In addition people with depression had worse subjective sleep quality and longer latency to REM sleep onset however no differences in other objective sleep quality measures were observed.

Our finding that older adults with lifetime depression have advanced patterns of Melatonin secretion is aligned with very early theories that people with major depressive disorder had advanced biological rhythms [45]. A small number of studies have shown individuals with affective disorders to have advanced circadian rhythmicity [46–48] with one reporting disruptions in melatonin secretion as well [46]. However, these studies had small sample sizes and to date findings from the current study represent the largest sample size of sleep and circadian assessment in older people with lifetime depression. Importantly, while it has been long documented that there are changes to the circadian clock during normal ageing including a reduction in amplitude of melatonin secretion and an advancement of phase [49, 50], our findings demonstrate that these are not due to normal ageing alone and are more pronounced in those with a history of affective disorder.

Conversely, a number of studies have also shown delayed melatonin secretion [31, 51, 52] but most of these latter studies have been in adolescent and young adult populations. One study in 72 older adults aged over 60 reported no significant associations between affective symptoms and melatonin secretion however only 55% had a lifetime history of affective disorder [53]. While in this study, we did not find that depression severity was associated with circadian misalignment, this may be due to a restricted range since our sample had largely sub-threshold symptoms. Certainly in symptomatic and samples under 60 years, prior studies have shown that an increased phase angle between melatonin onset and mid-sleep (in the delayed direction) was associated with severity of mood symptoms [54]. Overall, there is therefore emerging evidence for circadian disruption within affective disorders across the lifespan, which may differ somewhat according to age, depression subtype and symptom severity.

Importantly, despite 67% of our population using antidepressants, individuals with lifetime depression had a significantly longer phase angles than controls. There has been some evidence that treatment with antidepressants can phase realign the circadian clock [55, 56] particularly SSRIs. Of those using antidepressants only 26% were using SSRIs and therefore there was not an overall dampening effect. However, it is important to note, that there is an emerging literature suggesting that antidepressants increase sensitivity to light, which in turn, affects melatonin secretion [57] and circadian rhythmicity. In combination with external zeitgebers, activity, feeding patterns and other medical comorbidities, the effect of increased light sensitivity due to antidepressants could be pronounced, and could serve to either perpetuate circadian misalignment, sleep quality and timing, and depressive symptoms, or could play a vital role in symptom remission. Further research is required to ascertain the degree of light sensitivity in older people and antidepressant medications.

Consistent with prior research, we additionally found that subjective and aspects of objectively assessed sleep differed in those with lifetime depression. Poor sleep quality is in turn associated with poor cognitive functioning in both symptomatic and asymptomatic samples [58, 59] and brain connectivity in those with lifetime depression [60]. We found that those with depression had longer REM latency than healthy control participants. This could be attributed to a number of underlying mechanisms including alterations in the homeostatic drive for REM sleep as well as disturbances of the circadian system [61]. Alternatively, some antidepressant medications can suppress REM sleep and/or impact on latency [62]. In this study, 67% of participants with depression were taking antidepressants, however REM latency was not different between those who were and were not taking anti-depressants suggesting this was not the driving factor for this difference. The observed greater latency to REM in individuals with depression is in contrast to previous research, often focussing on middle-aged populations in which latency to REM sleep if often shortened. This could be due to an effect of ageing and emerging neurodegenerative pathology that may well be present in this population. We did not see any other significant differences in more traditional objective measures of sleep disturbance including wake after sleep onset and arousal index which is in line with other studies [63]. This suggests that circadian changes in older people with lifetime depression can occur without the concurrence of overt sleep disturbance.

An improved scientific understanding of the nature and mechanisms mediating the relationships between sleep and mood will ultimately assist in the delivery of more targeted and personalized treatments for this patient group. The need for this research is underscored by the fact that most antidepressant therapies actually alter sleep architecture, and can even exacerbate sleep disturbance, warranting interventions that are tailored to the individual sleep-wake profile [24]. Even though such research in its infancy, emerging data suggests that younger people with circadian phase delay may preferentially benefit from melatoninergic agents, aiming to advance the circadian rhythm [64]. Conversely, in older people, interventions aimed at specifically targeting sleep and the circadian system are required. Light therapy, physical exercise, sleep psychoeducation, cognitive activity, and transcranial magnetic stimulation have all been used as interventions aimed at realigning circadian rhythmicity, reducing nocturnal sleep disruption or enhancing sleep consolidation [65, 66]. Finally, the effects of antidepressants on sleep-wake systems cannot be under-stated. Further research exploring optimal maintenance treatments for those with remitted symptoms and persistent sleep-wake disruption is now needed. Older people with remitted symptoms and sleep-wake disturbance may be best suited to use of non-pharmacological treatments only such as psychological therapy [58]; non-REM suppressing medications, or those that are least disruptive to sleep architecture (e.g. Agomelatine*).*

We do need to acknowledge that this is a cross-sectional study and therefore we cannot draw causal inferences. Furthermore we studied a specific population with generally remitted depression symptoms and additionally had to exclude many other populations due to the nature of the outcomes and therefore our findings may not be generalizable to all older people with lifetime depression.

## Conclusion

Overall this study shows that in older people with lifetime depression and largely subthreshold symptoms, changes circadian functions are evident, compared to healthy controls, even when only small changes to sleep quality are apparent. Circadian disturbances may be possible targets of interventions to reduce depressive symptoms in older adults.

## Data Availability

The datasets used and/or analysed during the current study are available from the corresponding author at the discretion of the investigators.

## Abbreviations

DLMO: Dim light melatonin onset
HSO: Habitual sleep onset
PSG: Polysomnography
REM: Rapid-eye movement
SWS: Slow wave sleep
